# Isotope effects in dynamics of water isotopologues induced by core ionization at an x-ray free-electron laser

**DOI:** 10.1063/4.0000197

**Published:** 2023-10-03

**Authors:** R. Guillemin, L. Inhester, M. Ilchen, T. Mazza, R. Boll, Th. Weber, S. Eckart, P. Grychtol, N. Rennhack, T. Marchenko, N. Velasquez, O. Travnikova, I. Ismail, J. Niskanen, E. Kukk, F. Trinter, M. Gisselbrecht, R. Feifel, G. Sansone, D. Rolles, M. Martins, M. Meyer, M. Simon, R. Santra, T. Pfeifer, T. Jahnke, M. N. Piancastelli

**Affiliations:** 1 Sorbonne Université, CNRS, Laboratoire de Chimie Physique-Matière et Rayonnement, LCPMR, 75005 Paris, France; 2 Center for Free-Electron Laser Science CFEL, Deutsches Elektronen-Synchrotron DESY, Notkestr. 85, 22607 Hamburg, Germany; 3 European XFEL, 22869 Schenefeld, Germany; 4 Deutsches Elektronen-Synchrotron DESY, Notkestr. 85, 22607 Hamburg, Germany; 5 Lawrence Berkeley National Laboratory, Chemical Sciences, Berkeley, California 94720, USA; 6 Institut für Kernphysik, Goethe-Universität, 60438 Frankfurt am Main, Germany; 7 Department of Physics and Astronomy, University of Turku, 20014 Turku, Finland; 8 Molecular Physics, Fritz-Haber-Institut der Max-Planck-Gesellschaft, 14195 Berlin, Germany; 9 Department of Physics, Lund University, 22100 Lund, Sweden; 10 Department of Physics, University of Gothenburg, 412 96 Gothenburg, Sweden; 11 Physikalisches Institut, Universität Freiburg, 79104 Freiburg, Germany; 12 J. R. Macdonald Laboratory, Department of Physics, Kansas State University, Manhattan, Kansas 66506, USA; 13 Institut für Experimentalphysik, Universität Hamburg, 22761 Hamburg, Germany; 14 Department of Physics, Universität Hamburg, 22607 Hamburg, Germany; 15 Max-Planck-Institut für Kernphysik, 69117 Heidelberg, Germany

## Abstract

Dynamical response of water exposed to x-rays is of utmost importance in a wealth of science areas. We exposed isolated water isotopologues to short x-ray pulses from a free-electron laser and detected momenta of all produced ions in coincidence. By combining experimental results and theoretical modeling, we identify significant structural dynamics with characteristic isotope effects in H_2_O^2+^, D_2_O^2+^, and HDO^2+^, such as asymmetric bond elongation and bond-angle opening, leading to two-body or three-body fragmentation on a timescale of a few femtoseconds. A method to disentangle the sequences of events taking place upon the consecutive absorption of two x-ray photons is described. The obtained deep look into structural properties and dynamics of dissociating water isotopologues provides essential insights into the underlying mechanisms.

## I. INTRODUCTION

Water molecules exposed to x-rays can undergo structural modifications that are of utmost interest from both a fundamental point of view and with respect to the possible implications in a multiplicity of research fields. The relevance of radiation-induced dynamics ranges from radiation damage in living tissues^1^ to problems in oncology treatments^2^ and even interstellar phenomena.^3^ High-energy radiation can induce a variety of processes in water, such as the production of multiply charged species and fragmentation processes yielding ions or radicals. In particular, if a core electron is ejected, a relaxation process occurs, in which a valence electron fills the core vacancy, and another valence electron is emitted (Auger–Meitner decay). From gas-phase data, it is known that the resulting doubly charged water cation (H_2_O^2+^) eventually dissociates into fragments, which are mainly either a hydroxyl cation (OH^+^) or an atomic oxygen (O), and protons (H^+^).^4^ These dissociation processes have been studied by ion-mass spectroscopy^5^ or ion/ion-electron coincidence measurements.^4,6–9^ Core photoionization of water has been fully characterized in Ref. 10. Ultrafast fragmentation, i.e., bond breaking taking place within the core-hole lifetime, has been studied in water and heavy water following below-threshold photoexcitation^11^ and even in the O 1s ionization continuum of water^12^ by resonant Auger spectroscopy with synchrotron radiation as source. The fragmentation of water and its isotopologues ionized by short and intense laser fields have also been studied in various earlier works.^13–16^ For dicationic HDO, a remarkable isotope dependence in the production of OD^+^ vs OH^+^ has been found for the fragmentation of some dicationic states,^17–20^ which is attributed to a kinematic effect due to the different mass between proton and deuteron.^21^

In a recent study,^22^ conducted at the Small Quantum Systems (SQS) instrument^23,24^ of the European XFEL,^25^ we exploited the possibility of using very intense and very short x-ray pulses to induce sequential multiphoton absorption in isolated water molecules. We measured triple ion–ion–ion coincidences (H^+^/O^n+^/H^+^) and concentrated on the coincidence channel leading to two protons and a doubly charged oxygen ion, which can be almost entirely related to consecutive absorption of two photons and accompanying Auger–Meitner decay processes involving the O 1s inner shell. We stress that intermediate dicationic states probed this way are relevant in many situations where water molecules are exposed to x-rays or other high-energy radiation. Therefore, information on their dynamical evolution is crucial in a wealth of different fields of science.

By appropriate choice of experimental parameters and theoretical modeling, we demonstrated that with our approach, we can follow the dynamics occurring between two consecutive photoabsorption events in the time span of approximately 0–25 fs. We were able to characterize both structural and temporal evolution of the above-mentioned dissociation events, achieving a complete characterization of both geometrical changes and timing of the events and concerning all dicationic states formed after core ionization and Auger–Meitner decay.

In particular, we demonstrated that, on a timescale of a few femtoseconds after core ionization and subsequent Auger–Meitner decay, water undergoes structural deformation such as asymmetric O–H bond stretching and/or opening of the bond angle all the way up to 180°, eventually leading to rapid two-body or three-body fragmentation in asymmetric and/or unbent geometries.

In the present work, we extend the investigation to two water isotopologues, semi-deuterated water, HDO, and fully deuterated (heavy) water, D_2_O. Using the same instrumentation, data analysis, and theoretical modeling, we are able to investigate in unprecedented depth the related isotope effects. In particular, we show that similar bond elongation and bond-angle-opening mechanisms are present for all three isotopologues as well, exhibiting, however, significant differences. Specifically, the dynamical processes are slower in D_2_O and show pronounced asymmetries in HDO due to the heavier mass of D as compared to H. By combining the unique characteristics of the photon beam generated by the European XFEL, the coincidence imaging abilities of the COLTRIMS reaction microscope, and an advanced theoretical modeling, we show which deep insight we can obtain into a photoinduced dynamical process.

## II. METHODS

### A. Experiment

The experiment was performed at the Small Quantum Systems (SQS) scientific instrument^23,24^ of the European XFEL in Schenefeld, Germany.^25^ The accelerator was operated at 14 GeV electron energy with a bunch charge of 250 pC, and an effective number of 970 photon pulses per second was generated for the present experiment in the SASE3 soft x-ray undulator. The x-ray pulses had an estimated duration of about 10–25 fs and a maximum pulse energy of 4.4 mJ, measured after the last undulator.^23,24,26,27^ The photon energy was set to hν = 1000 eV, sufficient to extract all electrons from the investigated systems via consecutive photoionization and Auger–Meitner decay steps. The spectral bandwidth was determined as approximately 0.9% of the central photon energy. Data were collected using a COLTRIMS reaction microscope,^28^ with a supersonic gas jet consisting of a 50:50 mixture of H_2_O and D_2_O, leading to approximately 25% H_2_O/25% D_2_O/50% HDO vapor and helium. Experiments for gas jets containing pure H_2_O were performed as well. The water vapor was generated by heating a reservoir (located outside of the vacuum chamber) to 40 °C, while the gas line and the nozzle (consisting of an aperture of 200 *μ*m diameter) were heated to 50 and 70 °C, respectively. The jet was intersected with the x-ray beam. Charged particles that were generated in the interaction region due to the photoionization and decay processes were then guided to a position- and time-sensitive detector.^29^ From the measured position of impact of a particle and its time-of-flight, the particle's three-dimensional momentum vector was determined in an offline analysis. All ions generated in the photoreaction were measured in coincidence, which allowed to determine kinetic energies and relative emission angles. The detector employed for the measurement had an active diameter of 120 mm and consisted of a stack of two microchannel plates. The spectrometer used for guiding the charged particles to the detector consisted of an acceleration region with a strong electric field of E = 213 V/cm (with a length of 153 mm), followed by a region with constant electric potential. The overall distance between the interaction region and the ion detector was 250 mm. We refer to Ref. 22 for further experimental details.

For the results presented in Fig. 2, the measured ion momenta were transformed into a recoil frame of reference. This coordinate frame is defined by the emission direction of the O^2+^ ion (providing the x-axis of the coordinate frame) and the emission direction of one of the two emitted protons or deuterons. The latter spans the xy-plane together with the emission direction of the O^2+^ ion. The scatter plots shown in Figs. 3 and 4 are built in the same way as those reported in Ref. 22. A transformation similar to Fig. 2 was done for the results presented in Figs. 5 and 6, which uses a proton as the reference particle. In multicoincidence experiments, the detection of fragments that do not belong to the same parent molecule can lead to false coincidences that affect the quality of the data. In order to drastically reduce such events, the sum momentum of the three measured ions was inspected for each photoreaction event. After subtraction of the average linear momentum of the absorbed photons and the average molecular beam velocity, the sum reflects only the recoil momentum due to the emitted electrons. We have considered as valid only events where the sum momentum is less than <20 a.u. The overall ion rate observed in the experiment ranged from 1.3 up to 4.1 ions/photon pulse depending on the actual fluence of the XFEL light. Even for the highest ion rates, our sum momentum-filtering yielded a fraction of false coincidences of less than 7% in the final dataset.

### B. Simulation

The theoretical modeling of the multiple ionization and fragmentation dynamics has been described in Ref. 22. Here, we only give a brief overview of the methodology.

The dynamics of the water isotopologues was simulated using a kinetic Monte Carlo approach. We conducted *ab initio* molecular dynamics simulations, where, in each time step, we calculated on-the-fly potential energy gradients, photoionization cross sections, and Auger–Meitner decay rates for the current electronic state.^30–32^ While the molecular geometry was propagated, photoionization, fluorescence, and Auger–Meitner decay transitions took place randomly according to computed probabilities. The electronic structure was calculated using the XMOLECULE electronic structure toolkit,^30,32,33^ employing the 6–31G(d,p) basis set^34^ and the Hartree–Fock method. For open-shell configurations, we employed the restricted open-shell Hartree–Fock method.

The initial conditions for the molecular dynamics trajectories were sampled from the Wigner distribution of the vibrational ground state of the neutral molecule. A time step of 0.1 fs was used to propagate the molecular geometry, and the simulation was conducted for a total time of 200 fs. We sampled at a total number of 8000 trajectories.

For the x-ray pulse, we employed a photon energy of 1 keV and a temporal pulse shape described by two Gaussians, both centered at t = 90 fs, with full widths at half maximum of 5 and 35 fs and relative weights of 0.6 and 0.4, respectively. A total fluence of 2 × 10^11^ photons/*μ*m^2^ was employed. For a more detailed discussion of the pulse parameters, we refer to Ref. 22.

In order to disentangle the fragmentation dynamics of HDO, additional, more simplified simulations were performed for three model scenarios. In scenario 1 (instantaneous Coulomb explosion), the molecular geometry was propagated using bare Coulomb forces, where proton/deuteron carries a charge of 1 and oxygen a charge of 2. In scenario 2 (delayed ionization), also bare Coulomb force was used, but the oxygen atom was kept neutral for the first 10 fs. In scenario 3 (sequential fragmentation), on top of the delayed ionization of scenario 2, an artificial bond potential between O and D fragments was employed for the first 10 fs using bond parameters from the SPC/FW force field^35^ to mimic a sequential fragmentation.

## III. RESULTS AND DISCUSSION

### A. Newton diagrams

The coincidence channel leading to two singly charged light particles (protons or deuterons) and an oxygen ion with charge 2+ allows us to follow in detail the evolution of doubly charged water molecular ions formed by the absorption of a first photon and subsequent Auger–Meitner relaxation in a time interval in-between two consecutive photoabsorption events. Since it can be shown that the fluorescence yield for core-ionized oxygen is below 1%,^36^ the radiative decay channel is negligible.

**FIG. 1.**
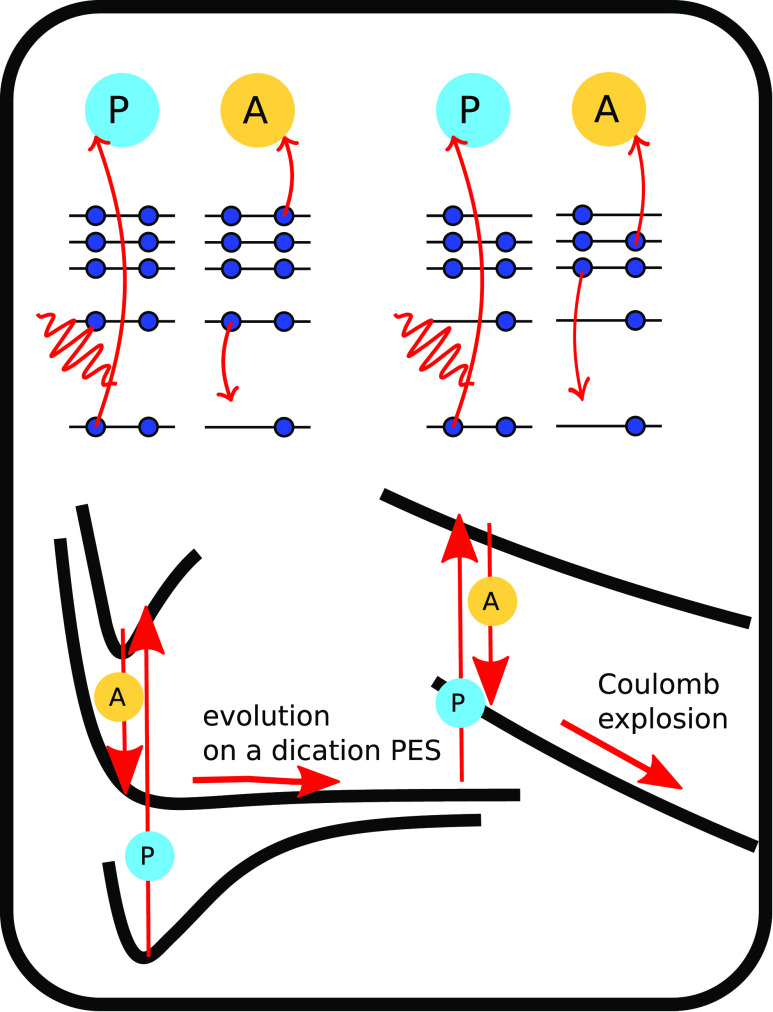
Top: photoionization and Auger–Meitner decay patterns induced by the consecutive absorption of two photons. Bottom: sketched evolution of the system on the different potential energy surfaces leading eventually to Coulomb explosion.

In Fig. 1, we show a schematic view of the processes, leading to the formation of the dicationic species and their evolution.

**FIG. 2.**
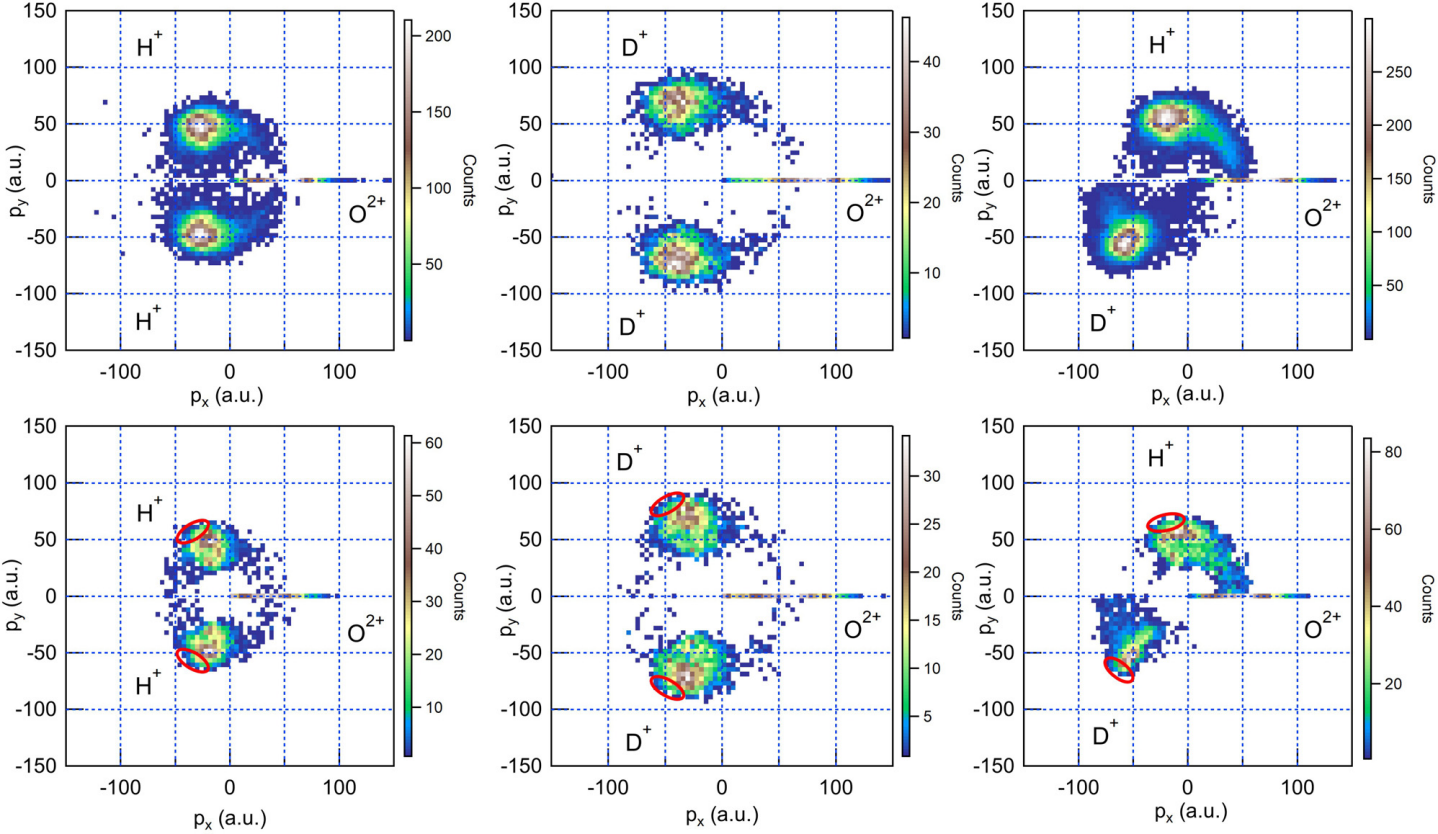
Newton diagrams (experiment, top and theory, bottom) of the ion momenta for O^2+^ and two H^+^ (D^+^) ions for H_2_O (left column), D_2_O (middle column), and HDO (right column), detected in coincidence. The oxygen momentum defines the x axis. In the theoretical plots, the red ovals mark the momentum values expected for an instantaneous Coulomb explosion from the neutral ground-state configuration.

In Fig. 2, we present the momentum space data for all three water isotopologues as Newton diagrams. The final momenta of the three ionic fragments O^2+^, H(D)^+^, and H(D)^+^ were detected in coincidence, as described earlier. The experimental diagrams are compared with simulations of the ionization and fragmentation dynamics conducted with the XMOLECULE toolkit.^30,32,33^

All three Newton diagrams show two dominant peaks for the H(D)^+^ momenta and a pronounced tail. The main peaks originate mostly from an almost instantaneous Coulomb explosion, caused by the absorption of two photons within a very short time interval: in all three systems, the final overall charge state 4+ is reached rapidly, and the intermediate molecular dication has not enough time to change its geometry. In contrast, the pronounced tail in the H(D)^+^ momentum distribution pointing toward the momentum direction of O^2+^ can be attributed to a situation where the delay between the two photoabsorption events is sufficiently large, allowing the molecule to undergo some form of structural rearrangement after the first photoionization event and first Auger–Meitner decay, and before it is torn apart by the strong Coulomb repulsion induced by the second photoionization event and second Auger–Meitner decay.

In our previous work,^22^ we demonstrated that the dynamical processes that can be related to the tail in the Newton diagram are of two different origins: an asymmetric bond elongation, whose limit is sequential two-body fragmentation into OH^+^/OD^+^ and D^+^/H^+^, and a bond-angle-opening trend, whose limit is a linear geometry or even an overbent configuration.

We now focus on the clear differences that we can immediately notice between the three Newton diagrams for H_2_O, D_2_O, and HDO.

In particular, the comparison between D_2_O in Fig. 2, middle column, and H_2_O in Fig. 2, left column, evidences a less pronounced tail in the former. This can be easily explained with the consideration that both motions we mentioned before, i.e., bond elongation and bond-angle opening, are slower in D_2_O due to the difference of mass between proton and deuteron. Therefore, during the same time interval, whose maximum length is set by the x-ray pulse duration, the percentage of molecular ions undergoing visible structural deformation is lower.

The comparison between H_2_O in Fig. 2, left column, and HDO in Fig. 2, right column, points to two effects. On the one hand, the angles between the momenta of the three particles are not symmetric, as is the case for both H_2_O and D_2_O. On the other hand, there is a tail for both H^+^ and D^+^ momenta toward the O^2+^ momentum, but the two tails are of different length and intensity.

The different angles between the H^+^ and D^+^ momenta with respect to the O^2+^ momentum (122° for the angle between H^+^ and O^+^ and 134° for the angle between D^+^ and O^+^ in momentum space) are simply a consequence of momentum conservation during the entire dissociation process. The peak values of the momentum distributions are close to the values one would expect from an instantaneous Coulomb explosion, marked as red ovals in the simulations (lower row in Fig. 2).

The difference in the tails of the momentum distributions in the Newton plots is more revealing of the different structural dynamics of the three ionized systems. The tail is clearly longer for H^+^, while the momenta of D^+^ are more concentrated toward the value corresponding to instantaneous Coulomb explosion. Due to the mass difference of the fragments, the momenta generated by the Coulomb explosion are not equally distributed across the three ionic fragments. In addition, the bond length between O and H increases more quickly than the O–D bond length. Accordingly, a dynamical process such as bond elongation is more advanced for the O–H bond when the second photon eventually triggers the final Coulomb explosion. This asymmetry is further enhanced by the subtle interplay of the two light atoms for some of the reached intermediate dicationic states. It has been reported that the lowest dicationic states in HDO asymptotically lead to a fragmentation into OH^+^ + D^+^ or OD^+^ + H^+^, where the latter is much more favorable by a ratio of 1:6^18,19^ due to the dynamical mass effect. If the delay between the two photoabsorption events is large enough, one may, thus, expect to see some intermediate stable DO^+^ and HO^+^ fragments with unequal ratio that are then ripped apart by the second fragmentation.

### B. Scatter plots

Additional information can be obtained by choosing a different representation of the data, which can give more detailed insight into the processes than the Newton diagrams, in particular on specific aspects of the molecular dynamics.

**FIG. 3.**
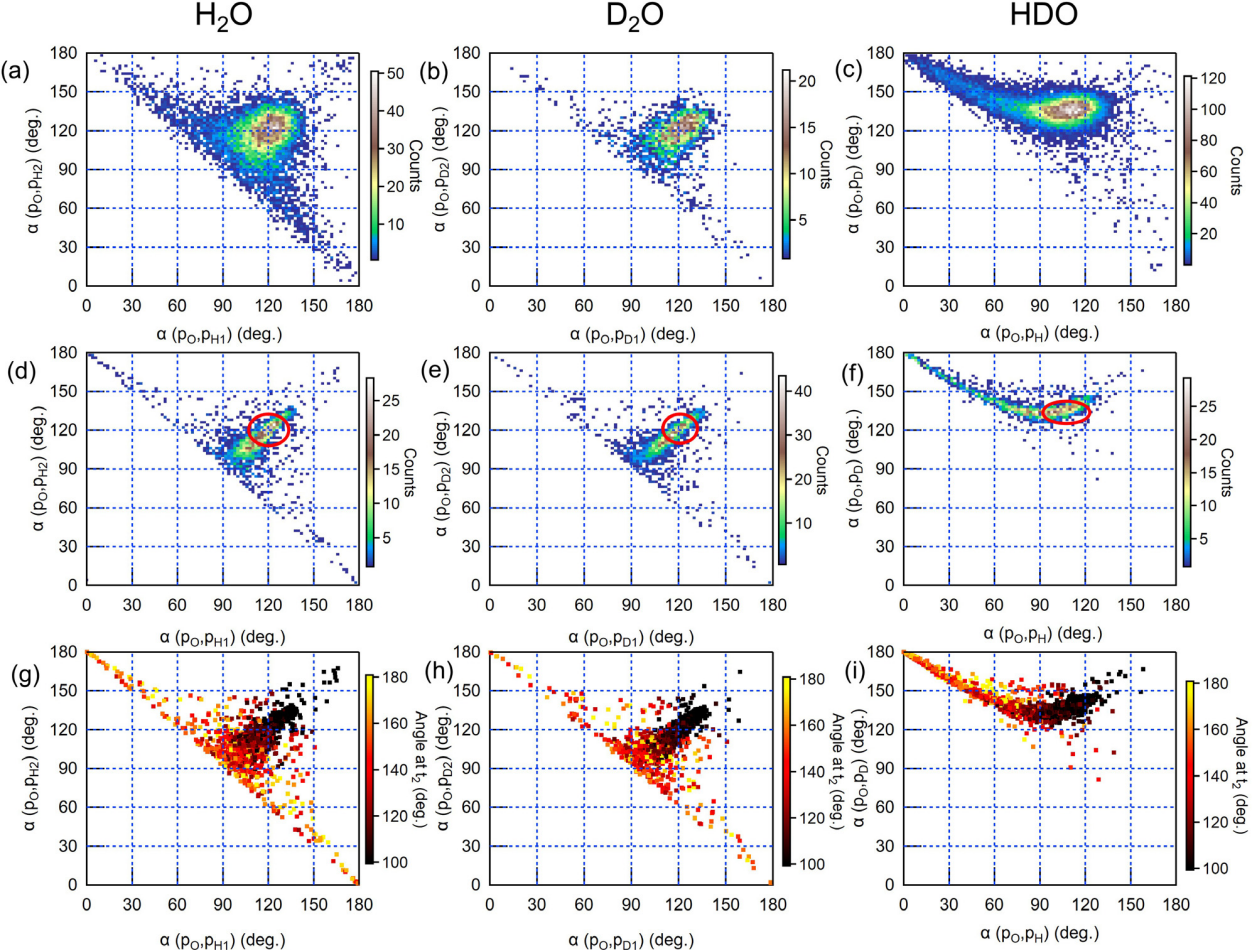
Scatter plots showing the angles of the final proton (deuteron) momenta with respect to the final oxygen momentum for H_2_O (left column), D_2_O (middle column), and HDO (right column). In each column, the top panel is the experiment, the middle panel is the simulation, and the bottom panel is the simulation where the color code represents the angles at the time of the second photoionization. The red ovals in the simulation panels indicate the value ranges for the event of a Coulomb explosion from the neutral ground-state configuration.

In Fig. 3, we, therefore, show proton/deuteron emission angles (experiment and simulations) with respect to the emission direction of the doubly charged oxygen ion for all three water isotopologues. From the correlation of the two angles shown in Figs. 3(a) and 3(d) (H_2_O, experiment and simulation) and Figs. 3(b) and 3(e) (D_2_O, experiment and simulation), it is clear that the majority of the emitted protons/deuterons is distributed at angles around ≈ 115°, which is close to what can be predicted for an immediate Coulomb explosion taking place in almost ground-state geometry (see the red ovals in the middle row of Fig. 3). However, in a significant number of events, the angles of the two protons/deuterons are strongly anticorrelated, and they exhibit an angle sum up to about 180°. In the simulations shown in Figs. 3(g) and 3(h), the events with large HOH-bond angle or DOD-bond angle, at the time of the second photon absorption are highlighted in orange and yellow, respectively. In our previous work,^22^ we discussed that this signal must be attributed to an unbending motion, where the water molecule reaches an almost linear geometry at this time when the second photoabsorption event triggers the final Coulomb explosion.

Comparing the simulation results with the experimental data, we find that the experimental data show a considerably wider distribution. We attribute this discrepancy to the recoil momentum imparted by the photo- and Auger–Meitner electrons on the ions, which is not taken into account in the simulation.

Earlier Coulomb explosion imaging experiments with intense optical laser fields found evidence for unbending motion of the water molecule.^14–16^ However, in optical experiments, it remains unclear whether only the removal of the electron or rather the subsequent driving of the remaining electrons or even re-collision effects are the main driver of structural dynamics.

An interesting observation in the present context is that this effect is clearly visible for both water and heavy water. No pronounced difference due to the heavier mass can be seen, which we attribute to the fact that we average over a larger variety of different delay times and intermediate dynamics. Overbending motion has been described in strong-field ionization of water^37^ and has been recently reported for D_2_O on a timescale of 20 fs,^9^ which confirms that it can take place within the maximum time interval of 25 fs available under our experimental conditions.

The comparison with HDO shows significant differences with respect to both H_2_O and D_2_O. The corresponding plots are shown in Figs. 3(c) (experimental), 3(f) (simulation), and 3(i) (simulation with color code indicating the angle at the time of the second photon absorption). The maximum density is centered around the values of the two angles that correspond to an instantaneous Coulomb explosion: ≈105° for H^+^ and ≈ 135° for D^+^. In addition, there is a tail toward larger angles for D^+^, up to a limit of 180°, and smaller angles for H^+^, down to a limit of 0°.

As mentioned before, the repulsion between O^2+^ and D^+^ gives a stronger kick to O^2+^ than the repulsion between O^2+^ and H^+^ does. Therefore, by a simple argument based on the difference of mass, the oxygen ion tends to be emitted back-to-back with respect to the deuteron. Furthermore, the O–H bond is likely to stretch more rapidly than the O–D one. In the limit of a molecule reaching a linear geometry after the absorption of the first photon and undergoing sequential fragmentation, the O–H bond is ruptured first, and the OD^+^ fragment undergoes Coulomb explosion upon arrival of the second photon. Therefore, the H^+^ and O^2+^ fragments move in the same direction, while the O^2+^ and D^+^ fragments move back to back. In the scatterplot, a whole distribution of angle combination values corresponding to all intermediate situations is visible.

**FIG. 4.**
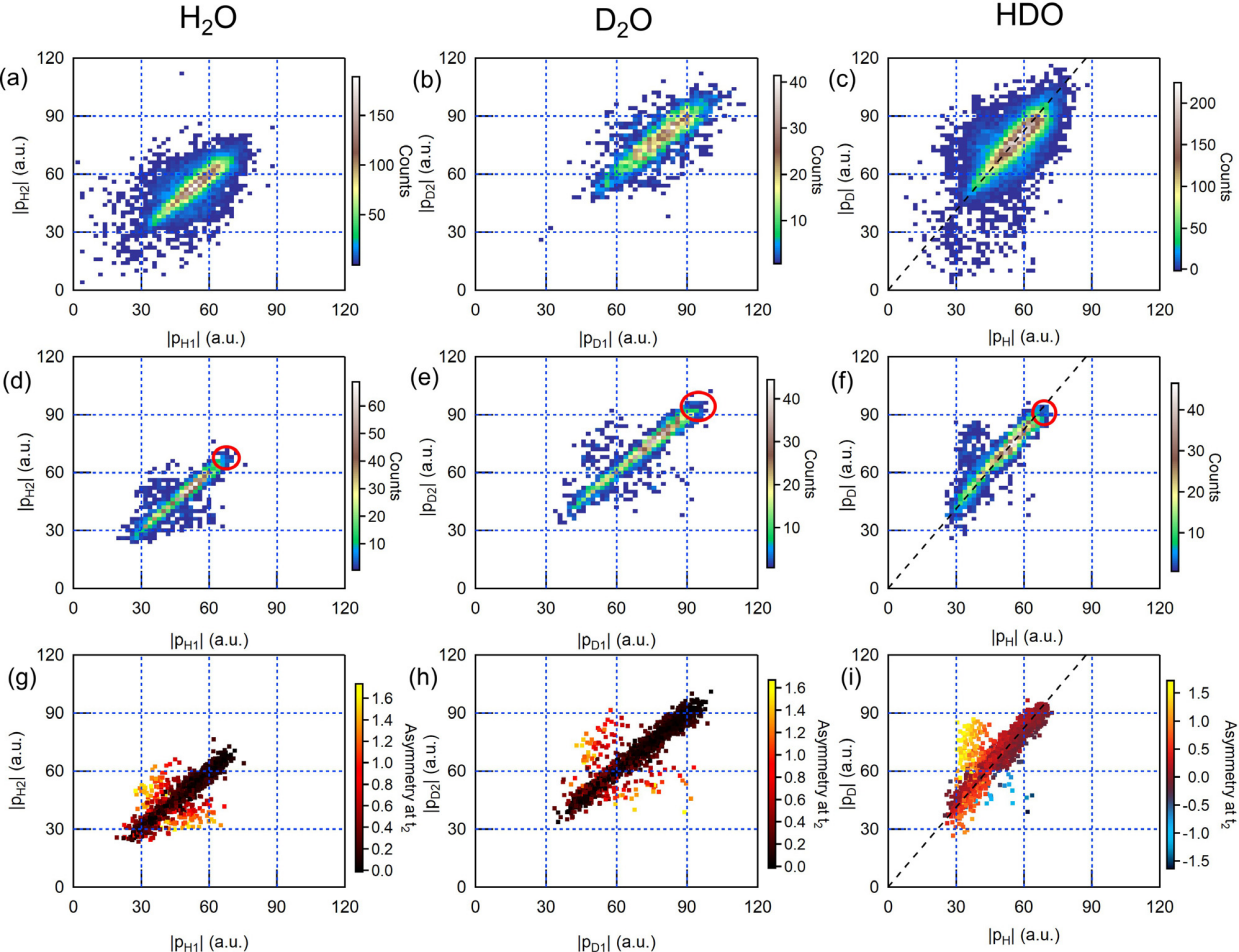
Scatter plots showing the magnitudes of the final proton/deuteron momenta for H_2_O (left column), D_2_O (middle column), and HDO (right column). In each column, the top panel is the experiment, the middle panel is the simulation, and the bottom panel is the simulation where the structural asymmetry at the time of the second photoionization is reflected by a color code. Asymmetry for H_2_O and D_2_O is defined as $2\left|d_{\mathrm{OX}1}-d_{\mathrm{OX}2}\right|/\left|d_{\mathrm{OX}1}+d_{\mathrm{OX}2}\right|$, where X stands for H and D, respectively. For HDO, asymmetry is given by $2(d_{\mathrm{OH}}-d_{\mathrm{OD}})/(d_{\mathrm{OH}}+d_{\mathrm{OD}})$. Thus, positive asymmetry indicates a breakup into OD^+^ + H^+^, whereas negative asymmetry indicates a breakup into OH^+^ + D^+^. The red ovals in the simulation panels indicate the value ranges for the event of a Coulomb explosion from the neutral ground-state configuration. The dashed lines in panels (c), (f), and (i) have a slope of 1.37 (see the text).

In Fig. 4, we show another type of scatterplot, displaying the absolute momenta of the two light ions. Figures 4(a) and 4(d) show the momenta for H_2_O, experiment and simulation, and Figs. 4(b) and 4(e) the momenta for D_2_O, experiment and simulation. Whereas most of the proton/deuteron momenta are clearly symmetric, i.e., they show similar absolute values, a significant fraction of them is asymmetric. In Figs. 4(g) and 4(h), the events in which the two particles have asymmetric OH or OD bond lengths at the time of the absorption of the second photon are shown in orange and yellow.

From this analysis, we can clearly assign asymmetric proton/deuteron momenta to asymmetric bond elongation after the first core photoionization and Auger–Meitner decay. Eventually, this asymmetric bond elongation may correspond asymptotically to a fragmentation channel of H_2_O^2+^ into OH^+^ and H^+^, which has been reported in the literature for the three lowest dicationic states^7,8,38–40^ and accounts for about 25%–30% of the total Auger–Meitner yield.^41,42^

The situation is again rather different for HDO. The absolute momenta are, in general, asymmetric, and most of them follow a linear relation. The slope of this linear relation can be understood if one considers the mutual Coulomb repulsion of O^2+^ and D^+^/H^+^ in a symmetric configuration independently, in which case

$$\frac{\left|p_{D}\right|}{\left|p_{H}\right|}=\alpha =\sqrt{\frac{m_{D}}{m_{H}}}\sqrt{\frac{m_{O}+m_{H}}{m_{O}+m_{D}}}≅1.37.$$ (1)

There are many points beyond this linear relation, suggesting a fragmentation from an asymmetric geometry. This interpretation is supported by the analysis of the simulation data in Fig. 4(i), where a color code indicates the relative interatomic distance $2(d_{\mathrm{OD}}-d_{\mathrm{OH}})/(d_{\mathrm{OD}}+d_{\mathrm{OH}})$ when the second photon is absorbed.

For the cases outside of the linear relationship, the deuteron momentum tends to be much higher than the momentum of the corresponding proton. Only for a few events, the proton momentum is significantly larger than the corresponding deuteron momentum. We quantify the degree of asymmetry by the fraction of data where

$$\left|\frac{\left|p_{D}\right|}{\left|p_{H}\right|}-\alpha \;\right|>0.2$$

and find a ratio of 0.3 (simulation) and 0.16 (experiment). As expected, the deuteration effect on the asymmetry found here is considerably smaller than reported before (1:6), which is related to the fact that we address here the overall yield of dicationic states and do not limit ourselves to the three lowest dicationic states. Nevertheless, the deuteration-induced asymmetry can be considered significant also for the overall Auger–Meitner yield.

### C. Native-frame analysis

In the discussion earlier, we have addressed the issues of asymmetric bond breaking and unbending motion, which are structural changes common to all three molecules under investigation.

While we can clearly identify an asymmetric bond elongation, from the aforementioned analysis based on Newton diagrams and scatter plots, it is difficult to clearly separate possible sequential steps. One of those could be a first complete bond rupture after the absorption of the first photon and then a Coulomb explosion of the remaining diatomic fragment after the absorption of the second photon. We can state that the complete sequential fragmentation is the limit case, and in the aforementioned figures, we see a distribution of all possible intermediate states.

In a previous work of some of the present authors, it was demonstrated that it is possible to distinguish sequential from concerted fragmentation in a triatomic molecule, CS_2_, on the ground of the different kinetic energy release (KER) ranges for the two processes.^43^ Furthermore, a method called “native-frame analysis” has been recently reported in the literature.^8,44–46^ The basic principle is to follow step-by-step a fragmentation process by creating “native frames” associated with each stage of the sequential dissociation. By using a combination of the momentum acquired by an undissociated ionic fragment in a first step and the momenta of fragments produced in a second step, it is possible to remove the momentum acquired in the first step and obtain the momentum distribution of the fragments produced in the second step, which is what is called “native frame” approach. It is an effective way to extract distinct information on the dissociation dynamics and to obtain the momentum distributions for one specific fragmentation process.

We concentrated our analysis on HDO, where the two chemical bonds are more likely to elongate and then break on different timescales. Assuming a two-step fragmentation where the O-H bond breaks and the proton leaves the molecule first, the final momentum distribution is a combination of the momentum acquired by the undissociated OD^3+^ fragment during the first step of the breakup, ***p***_OD_ = −***p***_H_, and the momentum acquired by the O and D ions during the second step, ***p*′**_O_ = −***p*′**_D_, in the center of mass frame of OD^3+^. Therefore, the final momenta ***p***_O_ and ***p***_D_ of the O and the D fragment after the Coulomb explosion can be written as

$$p_{O}={p\prime }_{O}-\frac{m_{O}}{m_{O}+m_{D}}\times p_{H},$$ (2)

$$p_{D}={p\prime }_{D}-\frac{m_{D}}{m_{O}+m_{D}}\times p_{H}.$$

Accordingly, the momenta acquired in the second step of an assumed two-step fragmentation (***p***′_O_ and ***p***′_D_) can be obtained from the final state momenta ***p***_O_ and ***p***_D_ by subtracting the recoil imparted by the emitted proton on the O and D ions in the first fragmentation step.

A first step along the native-frame-analysis scheme to shed further light on the sequential-vs-concerted dissociation is to plot the relative angle between the momenta of the H^+^ fragment and the momentum difference ***p***′_O_ - ***p***′_D_ as a function of the KER of the OD intermediate, calculated as

$$\mathrm{KER}\prime (\mathrm{OD})=\frac{{p_{\;O}^{\prime }}^{}}{2{\;m}_{O}}+\frac{{p_{\;D}^{\prime }}^{}}{2{\;m}_{D}}.$$

**FIG. 5.**
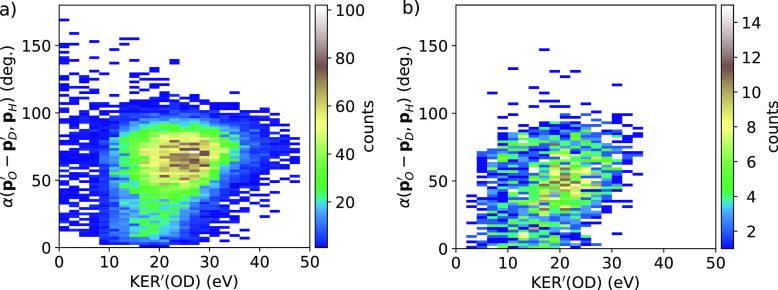
Angle between the momenta of the hypothesized OD^3+^ intermediate and H^+^ fragments vs intermediate KER for OD^3+^ in HDO (see equations in the text). Left: experiment. Right: theory.

We show such experimental and theoretical plots in Fig. 5. In the case of sequential ionization, we expect a broad distribution in the OD–H angle, up to covering the full range of 0°–180°. This would indicate full rotational turns of the OD intermediate after the emission of the proton. In contrast, if a sequential dissociation does not occur, or it is a minor channel, such angle would span a more limited range. This is actually the case in Fig. 5. These results indicate that the OD fragment is not free to rotate and hints at a predominantly concerted fragmentation process.

**FIG. 6.**
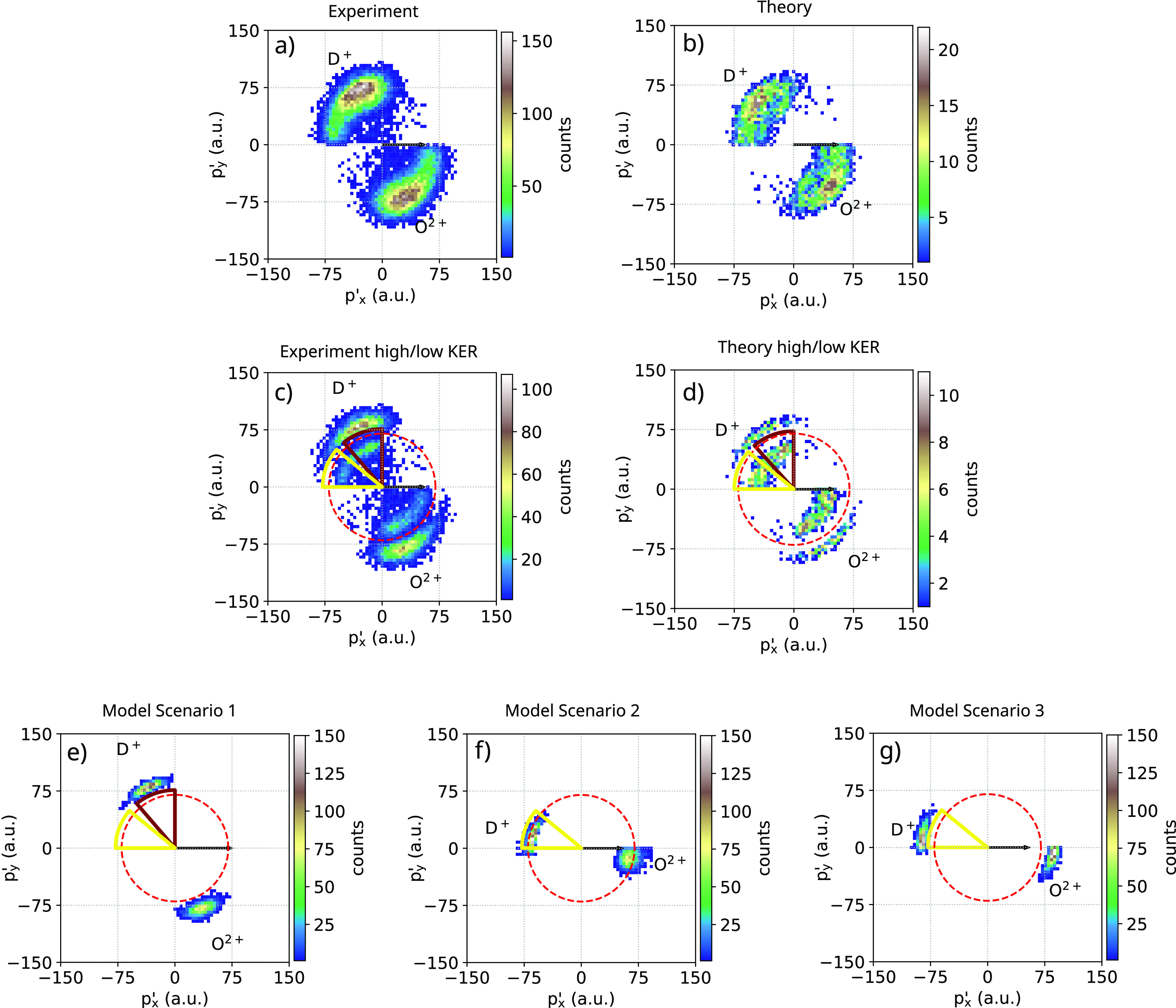
Native-frame plots for O^2+^/D^+^. The momentum vector of the H^+^ fragment is oriented along the horizontal axis. (a) and (b) Total momenta, experiment and theory; (c) and (d) corresponding plots, but gated on ranges of high and low KER. Here, low KER means KER < 30 eV, high KER means KER > 60 eV; (e)–(g) three different model scenarios (simulations) (see the text for details).

In Fig. 6, we show the momentum space results of the native-frame analysis of the HDO fragmentation, together with the theoretical simulations for three different possible scenarios. The corresponding plots are similar to the regular Newton diagrams shown in Fig. 2, but now, we depict the momenta of the two ions gathered in the second step of an assumed two-step fragmentation, i.e., the distribution of ***p***′_O_ and ***p***′_D_ in Eq. (2). In addition, we chose the momentum vector of the escaping H^+^ ion ***p***_H_ as the reference-axis, i.e., the x-axis in the figure.

In general, in a triatomic system, the momentum distribution associated with a sequential breakup is distributed along a circle, as the diatomic fragment left behind in the first breakup rotates with respect to the momentum vector of the departed atomic ionic fragment. The circle is typically considered as a clear sign of sequential fragmentation (see, e.g., the plot for the CS^2+^ fragment in CS_2_^45^).

In the plot of the experimental HDO data, Fig. 6(a), the momentum distributions align along quarter circle segments rather than a full circle. This seems to indicate again that the molecular dynamics do not consist of two independent dissociation steps, such as HDO^2+^ → OD^+^ + H^+^ after the absorption of a first photon, involving core photoionization and Auger–Meitner decay, followed by further fragmentation of OD^3+^ → O^2+^ + D^+^ after the absorption of a second photon, involving core ionization and a second Auger–Meitner decay in the OD^+^ fragment. We can identify nuclear motion, i.e., the structural deformation of the molecule, but no clear evidence of a clean sequential process. In other words, the features in the diagrams are linked to fragmentation after asymmetric/angular dynamics in the dication.

Our results may appear in contradiction with what is reported in the literature concerning a two-step dissociation in HDO, identified with a similar multicoincidence analysis,^8^ a theoretical investigation,^21^ and infrared laser pulses as source.^20^ However, we stress the point that in Ref. 8, the experiments are based upon one-photon double-valence photoionization, and there is no timing information concerning how long after photoionization the sequential breakup takes place. Similar considerations hold for the other works:^20,21^ the timing information is missing. As mentioned before, under our experimental conditions, we can follow all processes occurring between two photoabsorption events, within a maximum time span of about 25 fs (the estimated pulse duration). We gain the additional information that the sequential breakup (as identified by the native-frame analysis) “takes time,” and it is unlikely to occur on a very short timescale.

The same native-frame analysis was performed on the simulation data, Fig. 6(b), and by considering different limit cases based on pure Coulomb forces (details are given in Sec. II B).

In particular, in Figs. 6(c) and 6(d), we show the results from the experiment and the simulation by gating on a subset of the data excluding events with an intermediate KER of 30 eV < KER < 60 eV. Additionally, we marked some value ranges of the plot, which, in the bottom panel, correspond to the following different scenarios.

• Figure 6(e), model 1: instantaneous Coulomb explosion with charges O^2+^, H^+^, and D^+^;
• Figure 6(f), model 2: first dissociation due to Coulomb charges of O, H^+^, and D^+^, and then after 10 fs switch to O^2+^, H^+^, and D^+^, i.e., delayed photoionization;
• Figure 6(g), model 3: only release of H^+^ keeping OD^+^ connected via a harmonic spring, and after 10 fs breakage of the OD fragment into O^2+^ and D^+^, i.e., two-step fragmentation.

Model scenario 1, which corresponds to instantaneous Coulomb explosion, corresponds also to high momenta (and high KER), as already discussed concerning the Newton diagrams. Scenario 3 yields similar D^+^ and O^2+^ momentum magnitudes as in scenario 1. Compared to that, scenario 2 yields slightly lower D^+^ and O^2+^ momentum magnitudes. We can conclude that the main part of the features observed in the experiment, and the full simulation corresponds to what one sees in scenario 1: immediate Coulomb explosion with charges O^2+^, H^+^, and D^+^. The circle structure could be interpreted as a fragmentation of sequential nature, at least partially (extreme case, scenario 3, after 10 fs). Considering a continuum of delay times for both scenarios, the half-circle structure would emerge. However, also, just a delayed Coulomb explosion (extreme case, scenario 2: O neutral, H^+^, D^+^, and further charging up after 10 fs) gives a very similar picture.

We can conclude that in the present case, the native-frame analysis is effective in showing that the contribution of a pure two-step sequential dissociation (i.e., involving full rotations between the two fragmentation steps) is minor, at least in the time interval available, and different scenarios should also be considered.

### D. HDO dynamics

To better visualize the dynamical processes triggered by the photoabsorption and fragmentation processes of the HDO molecule, we now discuss snapshots from movies of the calculated trajectories. Those movies are available online.

**FIG. 7.**
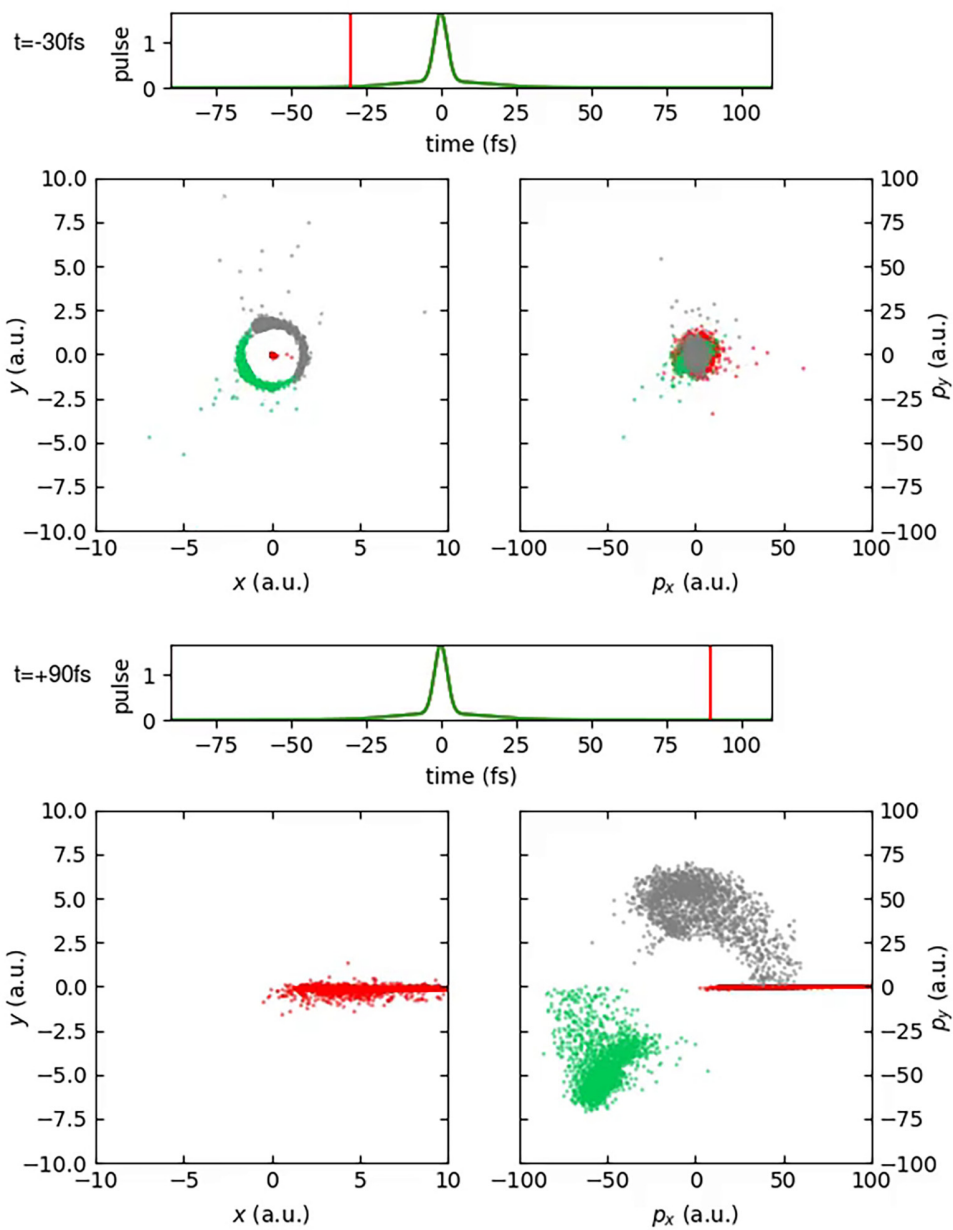
Position and momentum space coordinates in recoil frame for the ensemble of HDO molecules as a function of time. Left panels show position, and right panels show momentum space in the molecular plane. Gray points show the positions and momenta of hydrogen atoms, green dots show the positions and momenta of the deuterium atoms, and red dots show the positions and momenta of the oxygen atoms. The two horizontal “time” plots show the current time, and the green curve depicts the pulse envelope. Multimedia available online.
10.1063/4.0000197.1

Figure 7 (Multimedia view) shows the time evolution of the ensemble of HDO trajectories in position and momentum space. The coordinates are shown in the recoil frame, i.e., the frame where the final oxygen momenta are occurring along the x-axis. Snapshots are shown before (t = −30 fs) and after the center of the pulse (t = 90 fs). For the latter, the momentum coordinates are similar to the Newton diagram shown in Fig. 1, which represents the momentum coordinates in the recoil frame for t→∞. The earlier snapshot shows instead the HDO molecule having negligible momentum, but a broad distribution of orientations in position space. The hydrogen atom tends to point toward the upper right, and the deuterium atom tends to point toward the lower left direction.

**FIG. 8.**
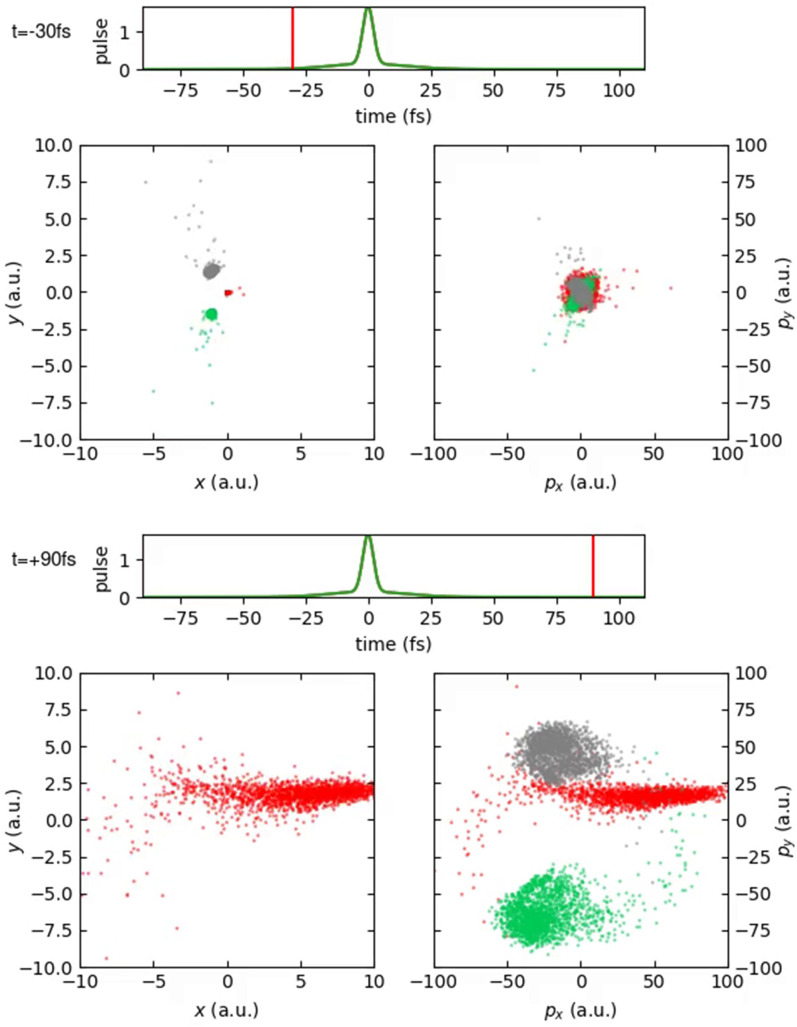
Position and momentum space coordinates in molecular frame for the ensemble of HDO molecules as a function of time. Left panels show position, and right panels show momentum space in the molecular plane. Gray dots show the positions and momenta of the hydrogen atoms, green dots show the positions and momenta of the deuterium atoms, and red dots show the positions and momenta of the oxygen atoms. The two horizontal time plots show the current time, and the green curve depicts the pulse envelope. Multimedia available online.
10.1063/4.0000197.2

In Fig. 8 (Multimedia view), the same evolution is shown in the molecular frame, i.e., in which the positions and momenta are sampled according to a Wigner distribution in the molecular rest frame. Here, the water molecule is initially oriented so that oxygen points on average to the positive direction.

The discrepancies between Figs. 7 and 8 are evident. At t = −30 fs, the position distribution resembles the geometry of the water molecule, whereas at t = 90 fs, the momenta show a different distribution than the Newton diagram.

The discrepancies between recoil and molecular frame arise largely due to the strong bending dynamics that have a noticeable impact on the final oxygen momentum and make a clear interpretation of Newton diagrams in Fig. 1 in terms of fragmentation dynamics challenging. As we also have reported before for H_2_O,^22^ in some cases, even overbending occurs, where the HOH angle grows beyond 180°.

Overall, it becomes visible that the dynamics induced by sequential core ionization involves large variations, caused by the different ionization timings and the different dicationic electronic configurations visited.

**FIG. 9.**
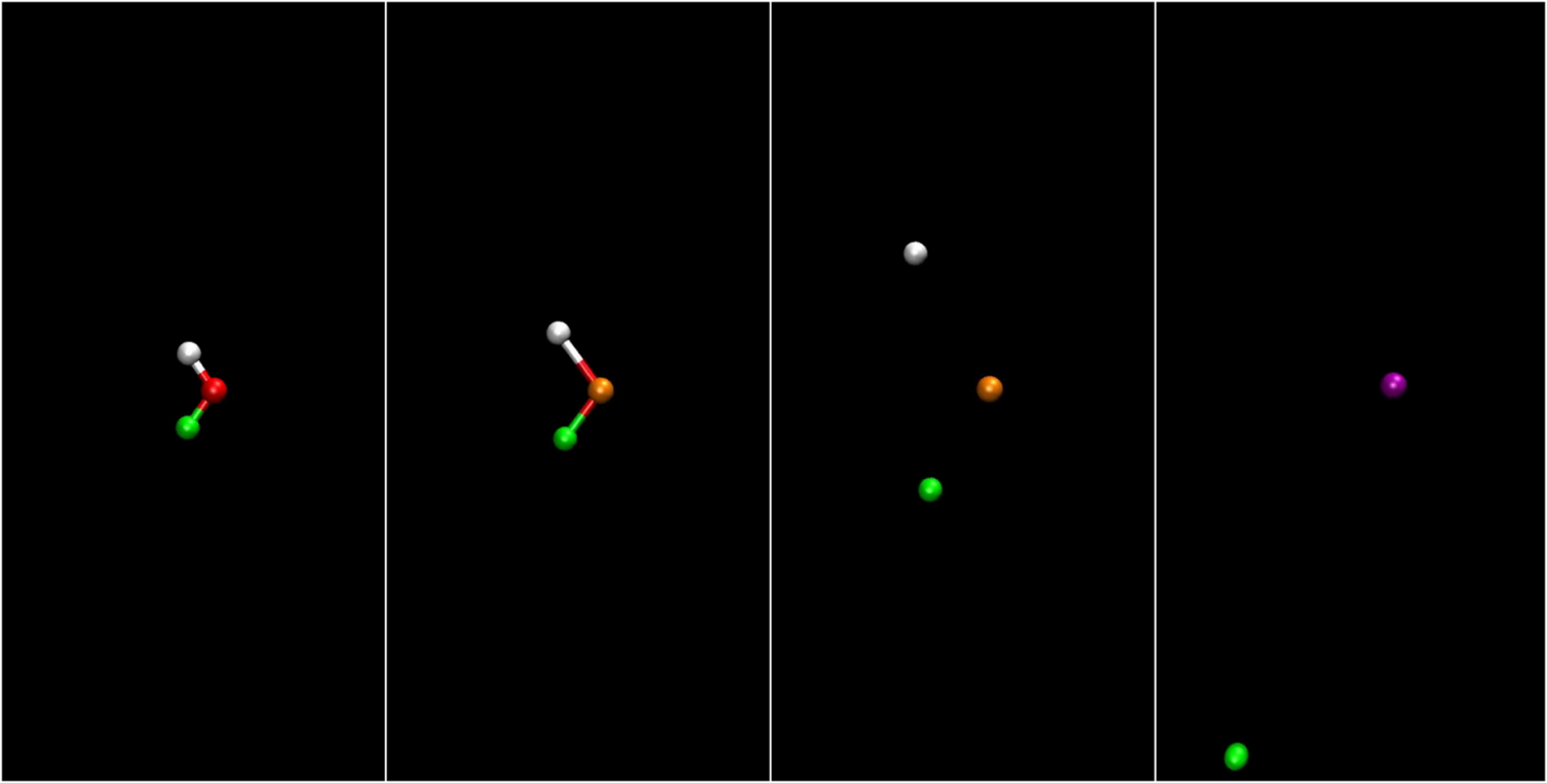
Selected trajectory with dissociation along bonds. White color denotes the hydrogen atom, green color the deuterium atom. The oxygen atom is depicted in red, pink, and orange colors, where the change in color denotes the timings of the two consecutive photon absorptions, namely, red: no photoabsorption, orange: absorption of the first photon, pink: absorption of the second photon. Multimedia available online.
10.1063/4.0000197.3

**FIG. 10.**
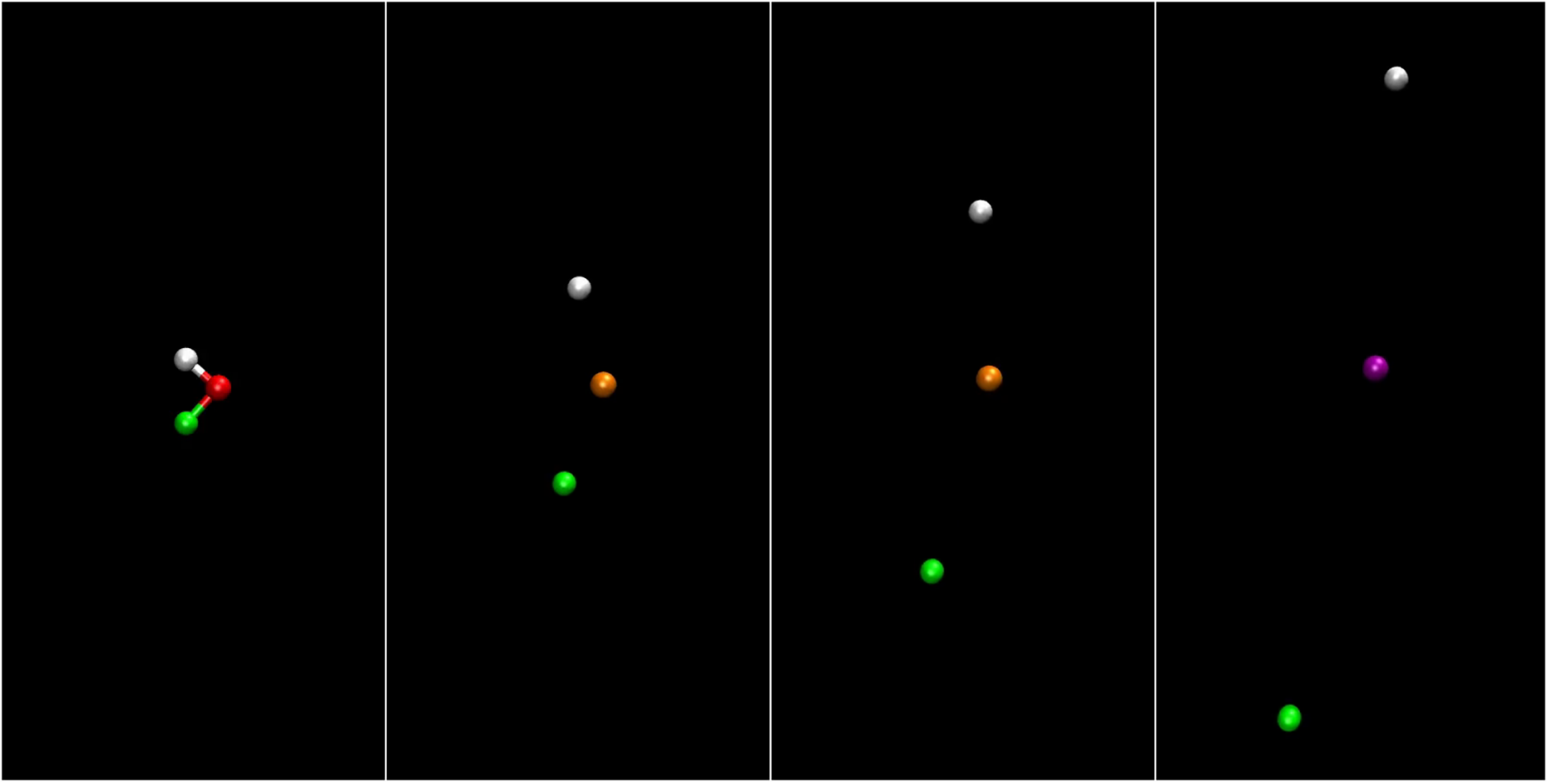
Selected trajectory with large unbending motion along the bonds. White color denotes the hydrogen atom, green color the deuterium atom. The oxygen atom is depicted in red, pink, and orange colors, where the change in color denotes the timings of the two consecutive photon absorptions, namely, red: no photoabsorption, orange: absorption of the first photon, pink: absorption of the second photon. Multimedia available online.
10.1063/4.0000197.4

**FIG. 11.**
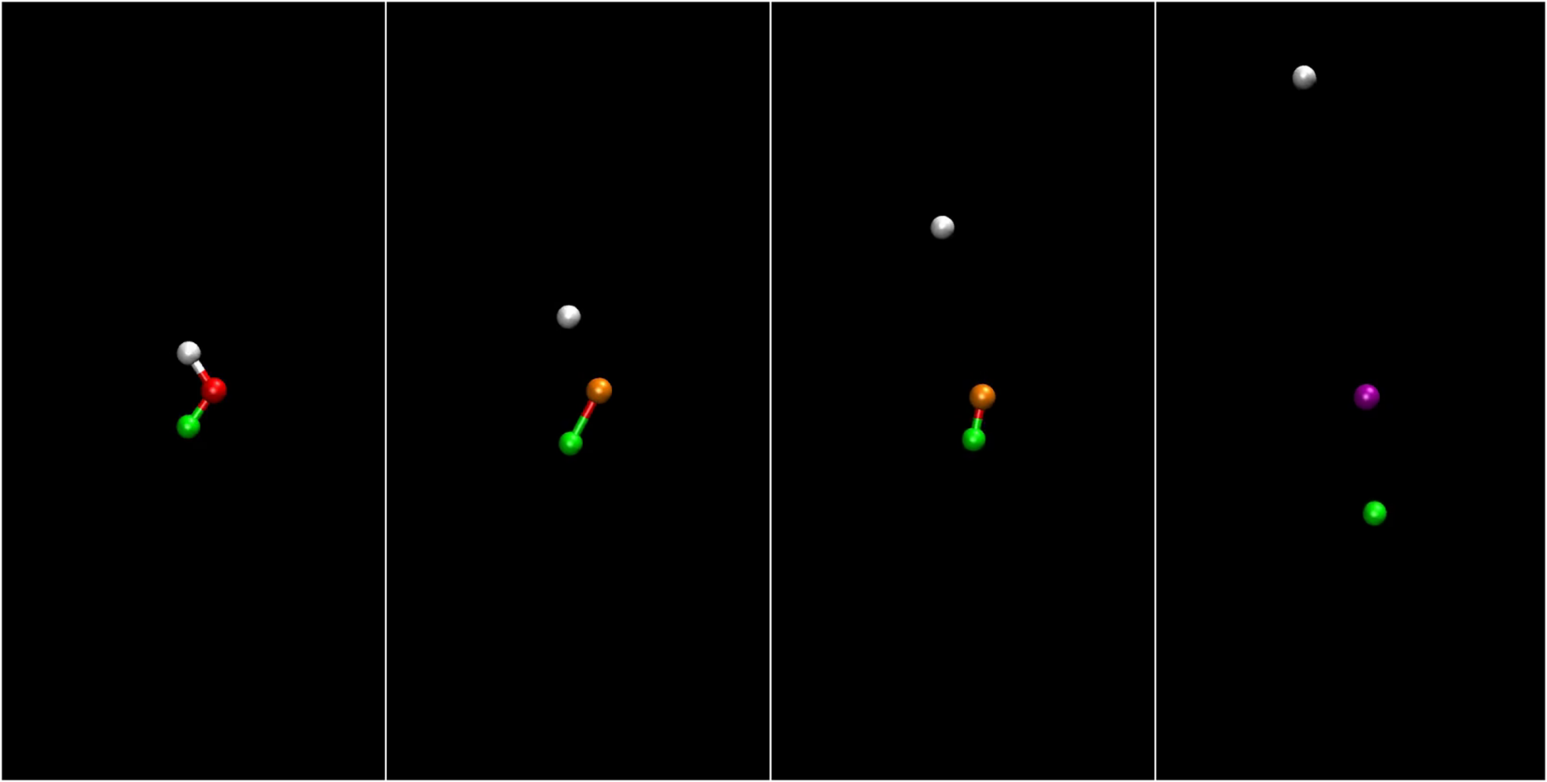
Selected trajectory with strongly asymmetric fragmentation. White color denotes the hydrogen atom, green color the deuterium atom. The oxygen atom is depicted in red, pink, and orange colors, where the change in color denotes the timings of the two consecutive photon absorptions, namely, red: no photoabsorption, orange: absorption of the first photon, and pink: absorption of the second photon. Multimedia available online.
10.1063/4.0000197.5

To further highlight this large variation, we selected trajectories out of the ensemble, where specific dynamical patterns show up in considerable strength. Figures 9–11 (Multimedia views) show trajectories where symmetric dissociation along the bonds, unbending motion, and strongly asymmetric fragmentation appears, respectively. The color change of the oxygen atom depicts the time at which photoabsorption occurs.

In Fig. 9, the first photoabsorption event triggers elongation of the OH and OD bonds and eventually dissociation along these directions into neutral O, D^+^, and H^+^. The second photoabsorption event then ionizes the oxygen atom and accelerates the fragmentation.

Figure 10 shows an example for strong unbending dynamics. Here, the three atoms reach an almost linear geometry when the second photon is absorbed, resulting in a back-to-back emission of D^+^ and H^+^ as discussed in Fig. 2.

The strong asymmetry displayed in Fig. 11 is an example for a quasi-sequential fragmentation. The first photoabsorption event triggers the release of H^+^, while the remaining OD^+^ fragment stays bound. Only after the second photoabsorption event, OD^+^ is ripped apart into O^2+^ and D^+^. This dynamical pattern leaves a significantly larger momentum on H^+^ than on D^+^, as highlighted in Fig. 4.

## IV. CONCLUSION

We have investigated the dynamics triggered by core-shell ionization in three water isotopologues, H_2_O, D_2_O, and HDO. We show in unprecedented depth isotope effects along the series. In particular, similar structural changes such as asymmetric bond elongation and bond-angle-opening mechanisms occur for all three systems, but with significant differences. Due to the larger mass, dynamical patterns are slower in D_2_O. Due to the asymmetry in mass for HDO, structural asymmetry arises in the dynamics. A method to identify the sequences of events taking place upon the consecutive absorption of two x-ray photons, the native-frame analysis, is also performed for HDO. It indicates that a clear two-step dissociation cannot be identified, possibly because a full sequential fragmentation takes more time than offered by the pulse duration of the ionizing x-ray beam of <25 fs. By combining the use of short and intense x-ray pulses with a detection method able to reveal structural changes in great detail, and including the insight obtained from an advanced theoretical modeling, we have shown differences and similarities in structural dynamics along a series of isotopologues and gained a deep insight into x-ray induced processes.

## Data Availability

The data that support the findings of this study are openly available in European XFEL at https://in.xfel.eu/metadata/doi/10.22003/XFEL.EU-DATA-002150-00, Ref. 47.
